# Medicated Soaps for the Treatment of Skin Disease

**Published:** 1851-03

**Authors:** 


*Medicated Soaps for the Treatment of Skin Disease.*—Sir H. Marsh uses the following prepartions in chronic forms of skin diseases:—

1. *Sulphur Soap.*—Take of

White Windsor Soap, 2 ounces;
Spirit of Wine, colored with alkanet root, 1 drachm.

Pound the soap in a mortar till all lumps have disappeared, add the spirit and beat into a paste, then add

Sulphur sublimed, 2 drachms;
Otto of rose, 10 drops;
Beat altogether.

2. *White Precipitate Soap*, as the above, substituting white precipitate for the sulphur.

3. *Red Precipitate Soap*, the same, substituting red precipitated mercury, 1 drachm.

4. *Corrosive Sublimate Soap*, the same, adding corrosive sublimate, 1 scruple.—*Dublin Med. Press.—Ib.*

